# Supplementary material for: A fast and sensitive room-temperature graphene nanomechanical bolometer
Source: Nat Commun. 2019 Oct 17;10:4726. doi: 10.1038/s41467-019-12562-2 (PMC6797740; doi:10.1038/s41467-019-12562-2)
Supplement: Supplementary file 1 — Supplementary Information [file 41467_2019_12562_MOESM1_ESM.pdf]

Supplementary Information

A fast and sensitive room-temperature graphene nanomechanical bolometer

[1,2,3]Andrew Blaikie, [1,2,3]David Miller, [1,2,3,4]Benjamín J. Alemán

[1]Department of Physics, University of Oregon, Eugene, Oregon 97403
[2]Material Science Institute, University of Oregon, Eugene, Oregon 97403
[3]Center for Optical, Molecular, and Quantum Science, University of Oregon, Eugene, Oregon 97403
[4]Phil and Penny Knight Campus for Accelerating Scientific Impact, University of Oregon, Eugene, Oregon 97403

*Corresponding Author: baleman@uoregon.edu

Supplementary Methods

Interferometric Transduction of Mechanical Motion. The motion of the graphene mechanical resonators was transduced with optical interferometry and lock-in amplification. Motion was actuated with a combination of a V_{DC} and V_{AC} electrical bias[1] between the graphene and the Si^{++} which produces a drive force, $F_{\mathrm{D}} \propto V_{\mathrm{DC}}\, V_{\mathrm{AC}} \cos \omega t$. A 633 nm probe laser (<1 μW) was focused down onto the graphene trampoline using a 40x, 0.6 NA objective. A low-finesse Fabry-Perot cavity, formed between the Si^{++} and the graphene, applies a small modulation to the reflected light as the resonator vibrates. We used a polarizing beam splitter and a quarter waveplate to split the reflected light from the incident beam. The intensity of the reflected beam was converted to a voltage using a silicon avalanche photodiode before being fed into a lock-in amplifier referenced to the applied V_{AC} electrical drive signal. The mode shape of the graphene drums[2] could be visualized by scanning the 633 nm probe laser across the device using a fast steering mirror with diffraction limited resolution. All measurements were done under vacuum at less than 10^{-5} Torr to minimize air damping. This optical setup is shown in Supplementary Figure 1.

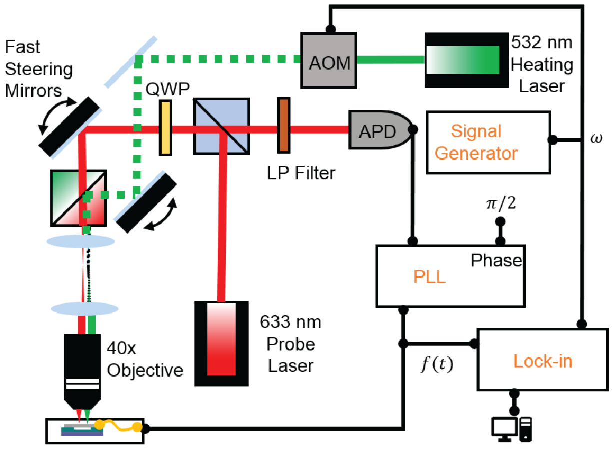

Supplementary Figure 1. Sketch of the optical interferometer setup used to measure the motion of and apply heating radiation to the suspended graphene.

Measurement of Frequency Shift Responsivity and Frequency Noise. To apply heating radiation, we used a 532 nm laser modulated with an acousto-optic modulator (AOM). The AOM was driven with a sine wave. A dichroic beam splitter was used to couple the heating laser into the optical path. We measured the incident power with a Thorlabs S120VC optical power meter just before entering the objective and estimate that the absorbed power is 2.3% of this value. When the suspended graphene absorbs light its mechanical resonance shifts. To track this resonance shift, we used frequency modulation[3] with a phase-locked-loop (PLL). The suspended graphene was electrically driven on resonance and the phase between the drive signal and amplitude signal was detected with a Zurich HFLI2 lock-in amplifier. The PLL feeds back on any deviation in phase by adjusting the drive frequency to keep this phase constant. The feedback bandwidth setting of the PLL was typically set between 1-50 kHz to not limit the frequency response. By reading out the adjusted drive frequency time series data, the PLL tracks any changes to mechanical resonance induced by the absorption of light or inherent frequency

fluctuations. This frequency time series data was then fit to a sine function with a frequency matching the AOM modulated frequency of the heating laser, with a modulated frequency between 40-100 Hz. To prevent any frequency drift due to substrate heating from interfering with the result, this fit was performed over many independent 100 ms time intervals and averaged. The amplitude obtained from the fit was used to calculate the frequency shift responsivity. Measurements of frequency noise were also performed with the PLL in the absence of heating radiation. This frequency noise time series data was used to calculate the Allan deviation[4], defined as

$$\sigma_{\mathrm{A}}^2 = \frac{1}{2(N-1)f_0^2}\sum_{\mathrm{m}=2}^{N}(f_{\mathrm{m}} - f_{\mathrm{m}-1})^2$$

where f_{m} is the average frequency measured over the mth time interval of length t.

Supplementary Note 1.

We characterized 12 devices for this work. Supplementary Figure 2 shows a gallery of SEM images of all the graphene nanomechanical bolometers (GNB) characterized. Supplementary Table 1 shows the mechanical and bolometric properties of these devices. The most sensitive noise equivalent power (device a) was calculated by measuring the Allan deviation at 1 ms, which we measured to be $\sigma_A = 1.9 \times 10^{-5}$.

	η (pW Hz$^{-1/2}$)	BW_T (kHz)	BW_{lw} (kHz)	R_f (W^{-1})	σ_A (10^{-5})	f_0 (MHz)	Q	V_{DC} (V)	w (µm)	d (µm)
a	7	11	6.4	300,000	2.1	16.6	2300	4	0.20	6
b	14	26	6.9	100,000	1.4	21.8	2700	-1	0.34	6
c	14	26	6.9	93,000	1.3	25.0	3100	-1	0.34	6
d	20	16	3.1	210,000	4.3	9.6	3400	1	0.52	8
e	22	14	10	180,000	4.1	10.7	910	0.25	0.50	8
f	25	12	4.8	260,000	6.5	11.1	2200	1	0.45	8
g	25	28	3.1	98,000	2.5	11.0	3600	1	1.4	8
h	41	65	5.0	39,000	1.6	25.4	4400	2	1.4	6
i	100	120	8.0	25,000	2.7	24.0	2600	-2	1.4	6
j	300	550	13	7,500	2.3	21.9	1400	-2	-	6
k	1100	1300	10	3,700	4.2	8.1	720	1	-	8
l	1500	1200	9.2	2,600	3.8	11.4	1100	1	-	8

Supplementary Table 1: Shows the detector sensitivity (η) at a 100 Hz bandwidth, the bandwidth estimated from the thermal response time (BW_T), the mechanical bandwidth (BW_m), frequency responsivity (R_f) to absorbed power, Allan Deviation (σ_A) over a 10 ms integration time, initial resonance frequency (f_0), mechanical quality factor (Q), gate voltage used for the measurements (V_{DC}), tether width (w), and initial diameter (d), for the 12 bolometers characterized in this work. Source data are provided as a Source Data file. Devices are indicated by a-l and correspond to the images in Supplementary Figure 2.

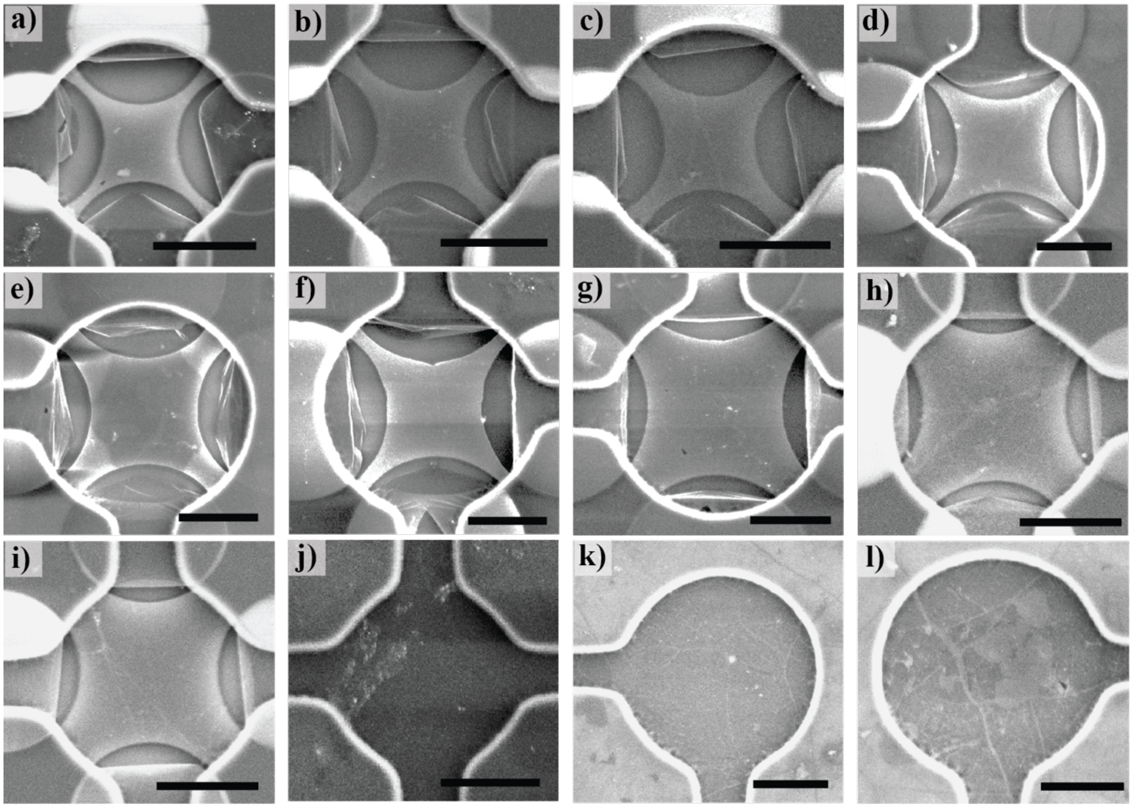

Supplementary Figure 2. SEM images gallery of all devices characterized. Devices are labeled by letters a-l. Devices a-i are trampolines and devices j-l are drumheads. Scale bars are 3 μm.

Supplementary Note 2.

Thermal Circuit. The response bandwidth and the overall response spectrum (Figure 3c in the main text) of our GNB are well described by a thermal RC circuit model, shown schematically in Supplementary Figure 3a. In this model, R_T is the combined thermal resistance of all four tether supports, C is the thermal capacitance given by the heat capacity of the suspended graphene, and I is the amplitude of absorbed power that is modulated at frequency ω. The surrounding support has relatively large thermal mass and is therefore assumed to be a thermal sink.

The effective thermal impedance of the circuit is

$$Z_\mathrm{T} = \left(\frac{1}{R_\mathrm{T}} + \frac{1}{\left(\frac{-i}{\omega C}\right)}\right)^{-1} = \frac{R_\mathrm{T}}{1 + i\omega R_\mathrm{T} C} = R_T \frac{1 - i\omega R_\mathrm{T} C}{1 + \omega^2 (R_\mathrm{T} C)^2}$$

The temperature difference between the graphene and the surrounding support is given by the thermal Ohm's law $\Delta T = I|Z_\mathrm{T}|$, or

$$\Delta T(\omega) = I \frac{R_\mathrm{T}}{\sqrt{1 + \omega^2 R_\mathrm{T}^2 C^2}} \qquad (1)$$

The characteristic time of the response is given by the thermal RC time constant:

$$\tau_T = R_T C.$$

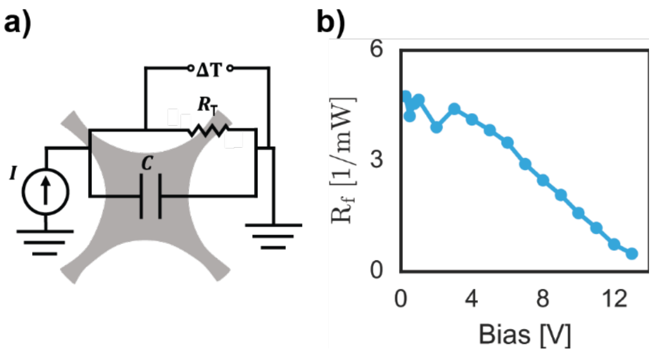

Supplementary Figure 3: Thermal circuit model and data. a) Schematic of the thermal circuit model used to model the bolometer performance. b) The frequency responsivity vs. bias voltage for the graphene trampoline. The bias voltage increases the stress on the trampoline which decreases its frequency responsivity. Source data are provided as a Source Data file.

Frequency-shift Responsivity. This ΔT will lead to a shift in the resonance frequency of the membrane. We relate ΔT to the frequency shift responsivity R_f by examining the change in resonance frequency (Δf_0) that results from thermal contraction of the graphene sheet. The equation for the mechanical resonance for a thin circular membrane is given by

$$f_0 = \frac{4.808}{4\,\pi\,r}\sqrt{\frac{\sigma}{\rho}}$$

where r is the radius, σ is the in-plane stress, and ρ is the 2D mass density. When the temperature of the suspended membrane increases, the stress changes according to the stress-strain relation, $\Delta\sigma = -(\alpha\,\Delta T)\frac{Y}{1-\nu}$, where Y is the Young's modulus, σ_0 is the initial stress, ν is the Poisson ratio, and α is the thermal expansion coefficient[5], which is negative for graphene for the temperature range used in our experiments. To first order,

$$\Delta f_0 = \frac{4.808}{4\,\pi\,r}\sqrt{\frac{\sigma + \Delta\sigma}{\gamma\,\rho}} - f_0 = f_0\left(\sqrt{1+\frac{\Delta\sigma}{\sigma_0}} - 1\right) \approx f_0\left(\frac{\Delta\sigma}{2\sigma_0}\right)$$

By the definition of R_f

$$R_f \equiv \frac{\Delta f_0}{f_0\,P_{\text{abs}}} = \frac{\Delta\sigma}{2P_{\text{abs}}\sigma_0}$$

or

$$R_f = -\frac{\alpha Y}{2P_{\text{abs}}\sigma_0(1-\nu)}\Delta T$$

Therefore, with Supplementary Equation 1,

$$R_f(\omega) = -\frac{\alpha Y}{2\sigma_0(1-\nu)}\frac{R_T}{\sqrt{1+\omega^2 R_T^2 C^2}} \tag{2}$$

We find excellent agreement between the model and the data for $R_f(\omega)$ (for example, see Fig. 3c in the main text). Moreover, the responsivity should be independent of incident power, in accord our measurements (see Fig. 2b in the main text).

Taking the thermal resistance as $R_{\text{T}} \sim \frac{\rho_{\text{T}} l}{w}$, where ρ_{T} is the 2D thermal resistivity, and l and w are the tether length and width, respectively, and in the limit $\omega \ll \frac{1}{R_{\text{T}}C}$, Supplementary Equation 2 becomes

$$R_f = \frac{\alpha Y \rho_{\text{T}}}{2\,\sigma_0(1-\nu)}\frac{l}{w} \tag{3}$$

The general $R_f \propto \frac{1}{w}$ prediction agrees well with the data (see Fig. 2c in main text.) We note that R_f is independent of the device area.

For a given device, R_f will decrease with added stress in the graphene. To check this prediction, we apply electrostatic stress with a back-gate bias V_{DC}, which pulls the graphene structure toward the silicon back-gate, while simultaneously measuring R_f. As predicted, we see that R_f decreases monotonically with increasing bias, as shown in Supplementary Figure 3b.

Noise-equivalent Power. The noise-equivalent power is defined as

$$\eta = \frac{\sigma_f\sqrt{t}}{\left|\frac{df}{dP_{\text{abs}}}\right|}$$

In principle, $\frac{df}{dP_{\text{abs}}}$ could be a function of P_{abs}, but in our system it is a constant. Thus,

$$\eta = \frac{\sigma_f\sqrt{t}}{f_0\left(\frac{\Delta f_0}{f_0 P_{\text{abs}}}\right)} = \frac{\sigma_{\text{A}}\sqrt{t}}{R_f}$$

where we have substituted the Allan deviation for fractional noise, $\sigma_{\text{A}} = \sigma_f/f_0$, and used the definition of the frequency-shift responsivity, $R_f \equiv \Delta f_0/(f_0 P_{\text{abs}})$. Using Supplementary Equation 3, η is given by

$$\eta = \sigma_{\text{A}}\sqrt{t}\,\frac{2\sigma_0(1-\nu)}{\alpha Y \rho_{\text{T}}}\frac{w}{l}$$

The predicted tether-width dependence $\eta \propto w$ agrees well across all devices tested in this work (Figure 2f main text), despite some variations in the Allan deviation. Thus, lower stress devices with a narrower tether width will have a lower noise-equivalent power (*i.e.* will be more sensitive to light.) As with R_f, η is independent of the device area.

Through narrowing the tether width w and increasing the tether length l, we expect a $R_{\mathrm{T}} \sim 10^{11}$ K W^{-1} (near the blackbody radiation limit[6]) to be within feasible experimental reach (the most sensitive devices characterized in this work have $R_{\mathrm{T}} \sim 10^{8}$ K W^{-1} see Supplementary Note 7). For our most sensitive GNB (device a), we would expect being able to access $\eta \sim 1$ fW Hz$^{-1/2}$, while still preserving a bandwidth of ~ 10 Hz. As a comparison, a 100 nm thick, 5 μm diameter bulk microbolometer with a heat capacity of $C \sim 10^{-10}$ J K^{-1} and $R_{\mathrm{T}} \sim 10^{11}$ K W^{-1} would have a bandwidth of ~10 mHz (see the following subsection Response Bandwidth), which would be over a $1000 \times$ slower than our GNB and impractical for most applications.

Response Bandwidth. In the thermal circuit model, $\tau_{\mathrm{T}} = R_{\mathrm{T}}C$ defines the 3-dB response bandwidth by $\mathrm{BW} = \frac{\sqrt{3}}{2\pi R_{\mathrm{T}} C}$. Taking the thermal resistance as $R_{\mathrm{T}} \sim \frac{\rho_{\mathrm{T}} l}{w}$ and $C = c\rho A$, where c is the membrane specific heat, ρ is the membrane mass density, and A is the membrane area, we have

$$\mathrm{BW} = \frac{\sqrt{3}}{2\pi c\rho\rho_{\mathrm{T}} l} \frac{w}{A}$$

The measured bandwidth data agrees well with model prediction BW $\propto w$ (see Fig. 3e in the main text). The BW we measure is likely lower than what we would expect for pristine graphene, as the mass density inferred from the resonance frequency gate dependence (see Supplementary Note 7) is about ~7.5 × greater than pristine graphene. Our experiments did not broadly sample the device area A, so we could not robustly test the prediction BW $\propto A^{-1}$. However, our limited data do agree with the area prediction. If the area dependence holds, the BW for a 1 μm diameter drumhead would reach ~40 MHz, and together with pristine graphene, the BW could reach ~200 MHz.

Finally, combining Supplementary Equation 3 with $\eta = \frac{\sigma_{\mathrm{A}}\sqrt{t}}{R_f}$ and $\mathrm{BW} = \frac{\sqrt{3}}{2\pi R_{\mathrm{T}} C}$, we obtain

$$\mathrm{BW} = \left(\frac{\sqrt{3}}{2\pi} \frac{\alpha_{\mathrm{T}}}{\sigma_{\mathrm{A}} \sqrt{t} c\rho} \frac{1}{A} \right) \cdot \eta$$

where $\alpha_{\mathrm{T}} \equiv \left(\frac{\Delta f}{f_0}\right) / \Delta T = -\frac{\alpha Y}{2\,\sigma_0 (1-\nu)}$ is defined as the frequency coefficient of temperature. The prediction $BW \propto \eta$ agrees well with our data, as shown in Figure 3f in the main text.

Mechanical Linewidth and Quality Factor. The mechanical damping time constant can be extracted from an amplitude frequency response curve (see Fig. 1d in main text). We use a model for a damped driven oscillator[7]

$$A^2 = I \frac{F^2}{(\omega_0^2 - \omega^2)^2 + 4\,\beta^2\,\omega^2}$$

where β is the 1/e decay rate, F is the driving force, $\omega = 2\pi f$, and $\omega_0 = 2\pi f_0$. We use this decay time to estimate the "mechanical bandwidth" from the resonance linewidth in the same way as from the thermal time constant, $\mathrm{BW_{LW}} = \frac{\sqrt{3}}{2\pi}\beta$. The quality factor is calculated according to $Q = \frac{\pi f_0}{\beta}$.

Supplementary Note 3.

Bandwidth Measurement using Resonant Frequency-Shift Response. We can measure the bandwidth of the GNB by increasing the modulation frequency of the heating laser and monitoring the response, thus directly measuring $R_f(\omega)$ and $\eta(\omega)$, and thus the bandwidth BW. While tracking the resonant frequency shifts with the PLL, we output a voltage proportional to the frequency shift from the PLL and input this into a second lock-in amplifier channel in the Zurich HFLI2. This signal is referenced to input of the AOM. By sweeping the frequency of the AOM drive signal we could quickly extract how the resonant frequency shift amplitude drops as the modulation frequency of the heating laser increases. Representative data for this measurement is shown in Fig. 3c in the main text. The 3dB point provides a direct measure of BW.

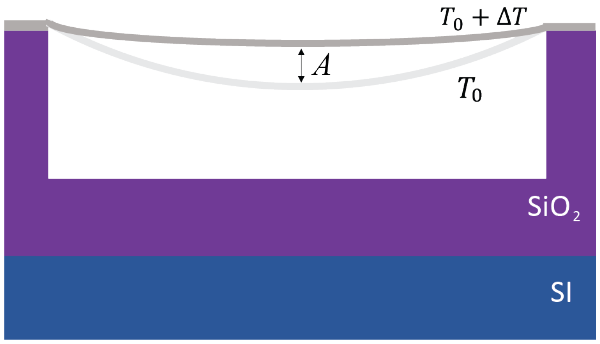

Supplementary Figure 4. Sketch of the off resonant GNB amplitude displacement, A, due to a change in temperature, ΔT.

Bandwidth Measurement using Off-Resonant Thermomechanical Response. We were unable to measure the bandwidth by looking at the change in frequency shift with the phase locked loop (PLL) when the device bandwidth exceeded that of the PLL. Instead, we infer bandwidth from the off-resonant frequency response of thermomechanical[8,9] displacement of the graphene membrane. The out-of-plane displacement of a curved membrane occurs when thermal stress tightens and flattens the membrane (Supplementary Figure 4). In the limit of small displacement and first-order thermal expansion, the mechanical displacement amplitude will be proportional to the change in temperature, $A \propto \Delta T$.

The displacement amplitude is a direct response to thermomechanical tensioning of the membrane, just as with the frequency-shift response of the bolometer. For the off-resonant

measurement, we modulate the heating laser at frequencies below mechanical resonance without applying any electrical actuation. The temporal response of the thermomechanical tensioning is expected to be the same in both the resonant frequency shift and the off-resonant amplitude response:

$$R_f(\omega) \propto A(\omega)$$

Thus, the off-resonant approach provides a good measure of the bolometer bandwidth. Solving the thermal circuit model (see Supplementary Note 2) yields the complex amplitude

$$A(\omega) \propto \frac{1 - i\omega\tau_{\mathrm{T}}}{1 + \omega^2\tau_{\mathrm{T}}^2}$$

where $\tau_{\mathrm{T}} = R_{\mathrm{T}}C$ is the thermal response time fit parameter and ω is the angular frequency of the heating source.

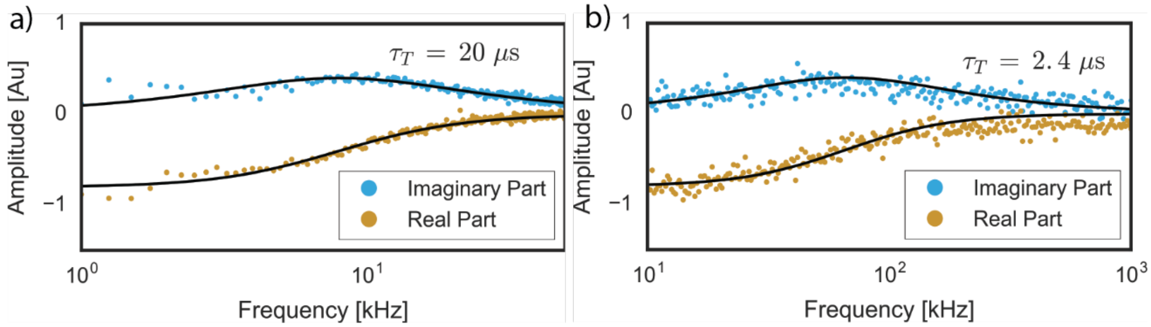

Supplementary Figure 5. Real and imaginary amplitude of thermal expansion induced displacement for two trampolines with different tether widths. These were fit to the thermal circuit model to extract the thermal response time, τ_T. **a)** Corresponds to device e in Figure 1. **b)** Corresponds to device i in Supplementary Figure 1. Source data are provided as a Source Data file.

In off-resonant experiments, we use the 532 nm heating laser to create an AC heat source and a 633 nm interferometer to measure the deflection of the graphene. The real and imaginary amplitudes (defined by the phase difference between the mechanical amplitude and the heating laser intensity) are shown in Supplementary Figure 5 for two different GNBs. The black traces are the model fits. The bandwidth can be obtained from the fitted thermal response time τ_{T} according to:

$$\mathrm{BW} = \frac{\sqrt{3}}{2\pi\,\tau_{\mathrm{T}}}.$$

We compared the response bandwidth obtained from the off-resonant and resonant approaches, denoted $\mathrm{BW_T}$ and BW, respectively. The selected results are shown in Supplementary Table 2 for devices e, f, and i (see Supplementary Figure 2). These results provide evidence that the BW obtained from the two approaches are equivalent, and that thermomechanical tensioning is

responsible for both off-resonant deflection and resonant frequency shifts. We also compared the BW to the equivalent bandwidth obtained from the mechanical resonance linewidth (denoted $\mathrm{BW_{LW}}$). A full comparison is provided in Supplementary Table 1. The results show that the response bandwidth ascertained from either the resonant or off-resonant approach is not determined or limited by the mechanical linewidth, as is expected[3]. For example, the bandwidth, $\mathrm{BW_T}$, for device l differs from the its linewidth bandwidth by over a factor of 100.

Device	$\mathrm{BW_T}$ from $A(\omega)$ (kHz)	BW from $R_f(\omega)$ (kHz)	$\mathrm{BW_{LW}}$ (kHz)
e	14	14	10
f	12	11	4.8
l	1200	--	9.2

Supplementary Table 2. This table shows the bandwidth estimated from the thermal response time ($\mathrm{BW_T}$), the mechanical bandwidth measured from the quality factor ($\mathrm{BW_m}$), and the 3dB bandwidth measured directly (BW). The direct bandwidth measurement agrees with the bandwidth limited by the thermal response time in agreement with frequency modulation.

Supplementary Note 4.

The figure of merit commonly used to compare different bolometers[10] is calculated using $\mathrm{FOM} = \mathrm{NETD} \times \tau_\mathrm{T} \times A_\mathrm{p}$ where NETD is the noise equivalent temperature difference and A_p is the detector area[11]. The NETD is proportional to the noise-equivalent power through the relation[11]

$$\mathrm{NETD} = \frac{4F^2\eta_\mathrm{p}}{\pi\, A_\mathrm{p}\left(\frac{\Delta L}{\Delta T}\right)\sqrt{t}}$$

where F is the optical aperture (typically $F = 1$), $\frac{\Delta L}{\Delta T} = 0.84$ W m^{-2} sr^{-1} K^{-1} is the luminance variation with scene temperature around 300 K, η_p is the noise equivalent power to incident radiation, and t is the measurement time. Increasing the thermal resistance improves η_p at the expensive of bandwidth and increasing the detector area improves the NETD at the expensive of pixel pitch. Therefore, the FOM removes geometric considerations when comparing bolometer technologies because both the thermal resistance and the pixel area can usually be tuned by changing the geometry. This is true for our GNB, as it is common to fabricate suspended graphene sheets with diameters ranging from $1 - 25$ µm (ref. [12]) and we have demonstrated that the thermal resistance can be tuned by varying the trampoline tether width. Doing this calculation for the most sensitive trampoline, with $\eta_\mathrm{p} = 300$ pW Hz$^{-1/2}$, $t = 10$ ms, $\tau_\mathrm{T} = 26$ µs, we obtain $\mathrm{FOM} = 1.18 \times 10^5$ mK ms µm^2. The lowest reported FOM for room-temperature microbolometers[10,11,13,14] is of order 10^5 mK ms µm^2. Thus, despite not yet being optimized and a low optical absorption (2.3%), our GNB has already matched these record-low FOM values.

Supplementary Note 5.

Optical Absorption Estimate from Cavity Effects. The GNB device architecture used in our studies forms a Fabry-Perot cavity. Optical cavity effects due to reflections at interfaces will lead to an optical absorption that is a function of the device dimensions and the wavelength of the absorbed light. We calculate the complex amplitude of the reflected electromagnetic wave from the Si and SiO_2 system at the location of the suspended graphene. Summing over all reflections according to the wave transfer matrix method for calculating transmission and reflection through multilayer media[15] gives the wave-transfer matrix

$$\mathbf{M} = \mathbf{M_B}(\mathrm{SiO_2} \to \mathrm{Si}) \times \mathbf{M_T}(\mathrm{SiO_2}) \times \mathbf{M_B}(\mathrm{vac} \to \mathrm{SiO_2}) \times \mathbf{M_T}(\mathrm{vac})$$

$$\mathbf{M} = \begin{pmatrix} A & B \\ C & D \end{pmatrix} = \begin{pmatrix} \frac{n_s + n_o}{2n_s} & \frac{n_s - n_o}{2n_s} \\ \frac{n_s - n_o}{2n_s} & \frac{n_s + n_o}{2n_s} \end{pmatrix} \times \begin{pmatrix} e^{-i\frac{2\pi n_o d_o}{\lambda}} & 0 \\ 0 & e^{i\frac{2\pi n_o d_o}{\lambda}} \end{pmatrix} \times \begin{pmatrix} \frac{n_o + 1}{2n_o} & \frac{n_o - 1}{2n_o} \\ \frac{n_o - 1}{2n_o} & \frac{n_o + 1}{2n_o} \end{pmatrix} \times \begin{pmatrix} e^{-i\frac{2\pi d_v}{\lambda}} & 0 \\ 0 & e^{i\frac{2\pi d_v}{\lambda}} \end{pmatrix},$$

where $\mathbf{M_B}$ describes a dielectric boundary reflection and $\mathbf{M_T}$ describes the propagation through a homogeneous medium. We calculate $\mathbf{M}$ by using standard values for the refractive index of SiO_2 and Si ($n_o = 1.5$ and $n_s = 4.14$) and by measuring the thickness of the oxide layer and the distance between the graphene and the oxide with atomic force microscopy (see Supplementary Figure 6 for schematic displaying d_o and d_v), from which we obtain $d_o = 353$ nm and $d_v = 552$ nm, respectively, and use $\lambda = 532$ nm.

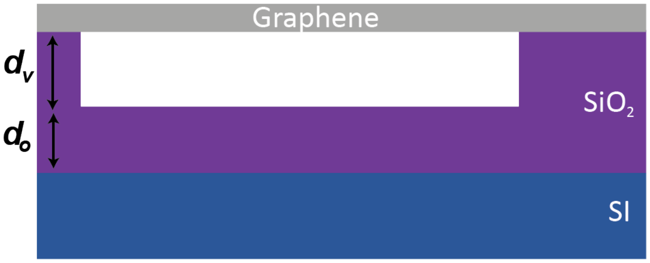

Supplementary Figure 6. Sketch of the GNB where d_v is the distance between the suspended graphene and the SiO2 and d_o is the oxide layer thickness protecting the silicon back gate.

In terms of the wave-transfer matrix components C and D, the amplitude of the complex reflected wave is $u_r = -\frac{C}{D} = (-0.55 + 0.26\, i)$. The electric field intensity at the graphene can be written as the sum of the incident and reflected wave $|u|^2 = |1 + u_r|^2$. The power absorbed by the graphene can be written as a function of electric field intensity at the graphene because the reflection coefficient of graphene is small ($r \sim 0.01$)[16],

$$\beta = \pi\alpha|1 + u_r|^2 = 0.6\%$$

where β is the absorption coefficient. We note that an engineered cavity could be used to enhance the absorption to $\beta = \pi\alpha|1+1|^2 = 9.2\%$.

Supplementary Note 6.

Photothermal Back-action Frequency Responsivity. Here we estimate the frequency shift due to photothermal backaction[16]. Depending on the location of the graphene membrane in the optical field of the cavity, this photothermal backaction could either enhance or weaken the GNB thermomechanical responsivity. Our modeling and calculations show that photothermal backaction does not cause a significant frequency shift in a GNB when compared to photothermal tensioning.

The effective frequency ω_{eff} and damping Γ_{eff} due to photothermal backaction can be written as[16]

$$\omega_{\text{eff}} = \omega_0\left(1 - \frac{1}{1+\omega_0^2\tau^2}\frac{\nabla F}{K}\right)^{\frac{1}{2}}$$

$$\Gamma_{\text{eff}} = \Gamma\left(1 + Q\omega_0\tau\,\frac{1}{1+\omega_0^2\tau^2}\frac{\nabla F}{K}\right)$$

where τ is the thermal response time, $\nabla F = \frac{dF_{\text{pth}}}{dz}$ is the derivative of the photothermal force as a function of displacement (ref. [16]), and $K = m\omega_0^2$ is the effective spring constant. Approximating ω_{eff} with a series expansion, because $\nabla F/K \ll 1$ for low absorbed powers, we obtain

$$\omega_{\text{eff}} \approx \omega_0\left(1 - \frac{1}{2(1+\omega_0^2\tau^2)}\frac{\nabla F}{K}\right) = \omega_0 - \frac{1}{2}\left(\frac{\frac{\Gamma_{\text{eff}}}{\Gamma} - 1}{Q\tau}\right)$$

Then, the frequency responsivity due to photothermal backaction, given by $R_{f,\text{BA}} = \frac{\omega_{\text{eff}}-\omega_0}{\omega_0 P_{\text{abs}}}$, is the relative change in the effective resonance frequency with respect to absorbed power, is

$$R_{f,\text{BA}} \approx \frac{1}{2}\left(\frac{1}{Q\omega_0\tau}\frac{\Gamma_{\text{eff}}/\Gamma - 1}{P_{\text{abs}}}\right)$$

In this work, we did not have a back-reflecting mirror to enhance cavity effects. Therefore, we use the measured results from Ref. [16] and assume an optimized photothermal backaction setup to estimate the upper limit of the frequency responsivity due to photothermal back-action. We use $\frac{\Gamma_{\text{eff}}/\Gamma-1}{P_{\text{abs}}}$~2 mW^{-1}, Q~500, P_{abs}~$P_{\text{inc}} \times 0.023$, $\omega_0 = 2\pi \times 5$ MHz, and we estimate τ~300 ns instead of the theoretical estimate provided in Ref. [16] to account for the slower than theoretically predicted thermal response time in suspended graphene[8]. The τ~300 ns estimate used here is consistent with the thermal response time from our measurements and that of Dolleman et al.[8] Altogether, we estimate $R_{f,\text{BA}}$~10^2 W^{-1} for a 10-micron drumhead with a back-reflecting mirror. Therefore, the change in frequency due to photothermal back-action, even assuming a perfect

reflecting back mirror, is a factor of $10^2 - 10^4$ lower than direct photothermal tensioning (see Supplementary Table 1 for R_f.) Moreover, because for a given absorbed power and displacement the device heats up to a higher temperature resulting in a larger photothermal force—and thus $\nabla F \propto R_{\mathrm{T}}$— any enhancement to ∇F due to an increased R_{T} would be canceled by an increase in thermal response time, where $\tau \propto R_{\mathrm{T}}$. Consequently, the trampolines, which possess a larger R_{T}, would see little if any enhancement in photothermal backaction when compared to the drumheads.

Supplementary Note 7.

Resonance Frequency Gate Dependence. The resonance frequency gate dependence ($f(V_{\mathrm{DC}})$)—as seen in Supplementary Figure 7—can be used to infer[17] the graphene membrane mass density (ρ), Young's modulus (Y), and initial stress (σ_0). In turn, these quantities provide independent ways to experimentally calculate C, R_{T}, R_f, and the absorption β. The relevant equations are derived from the capacitive potential energy of the graphene sheet and the Si^{++} back-gate.

Referring to the schematic given in Supplementary Figure 6, the series capacitance of the vacuum and oxide is

$$\frac{1}{C_{\mathrm{e}}} = \frac{d_{\mathrm{v}}}{\epsilon_0 A} + \frac{d_o}{\epsilon_0 \epsilon_{\mathrm{r}} A} = \frac{d}{\epsilon_0 A}$$

where A is the area of the drumhead and $\epsilon_{\mathrm{r}} = 3.9$ is the relative permittivity of SiO_2, d_{v} is the distance between the suspended graphene and oxide (which is in vacuum), and d_{o} is the thickness of the oxide between the Si^{++} and vacuum, and $d = d_{\mathrm{v}} + \frac{d_{\mathrm{o}}}{\epsilon_r}$. The capacitive potential energy is

$$U_{\mathrm{C}} = \frac{\epsilon_0 V_{\mathrm{DC}}^2}{2} \int \frac{dA}{d_{\mathrm{v}} + \frac{d_{\mathrm{o}}}{\epsilon_{\mathrm{r}}} - x}$$

where x is the displacement of the suspended membrane, and V_{DC} is the applied DC gate bias. We then use U_{C} in the Lagrangian procedure outlined in Ref. [17] to obtain

$$(2\pi f)^2 = \frac{2.4048^2 \sigma_0}{r^2 \rho} - \frac{\epsilon_0 V_{\mathrm{DC}}^2}{d^3 \rho} + 0.1316 \frac{Y\, \epsilon_0^2 V_{\mathrm{DC}}^4}{(1-\nu^2)\rho\, \sigma^2} \tag{4}$$

$$\sigma = \sigma_0 + \frac{Y\, r^2 \epsilon_0^2 V_{\mathrm{DC}}^4}{(1-\nu^2)\, 128\, d^4 \sigma^2} \tag{5}$$

where r is the radius of the drumhead, ϵ_0 is the permittivity of free space, and $d = d_\text{v} + \frac{d_\text{o}}{\epsilon_\text{r}}$ is the effective capacitive distance, which we measure from atomic force microscopy to be $d = 642$ nm.

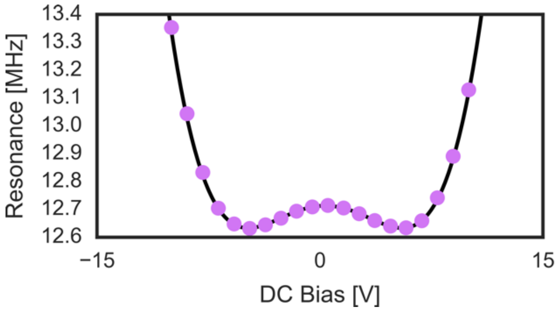

Supplementary Figure 7: The resonance frequency plotted vs. bias voltage for device l (see Supplementary Figure 1). To extract the resonance frequencies, amplitude-frequency response curve data was fit using damped driven oscillator at varying gate voltages. Using these values, the mass density, initial stress, and elastic modulus were extracted. Source data are provided as a Source Data file.

Fitting the experimental data of $f(V_\text{DC})$ for a given membrane device using Supplementary Equations 4-5 yields the parameters ρ, Y, and σ_0. Using the data for the drumhead device l shown in Supplementary Figure 6, we extract $\sigma_0 = 0.1$ N m^{-1}, $\rho = 7.5 \times \rho_\text{g}$, and $Y = 110$ N m^{-1}, where ρ_g is the intrinsic mass density of monolayer graphene ($\sim 7.7 \times 10^{-7}$ kg m^{-2}). The amount of contaminating mass observed in this device is consistent with other graphene nanomechanical systems that used a PMMA transfer technique to suspended graphene sheets[16,18]. We expect that the mass density and modulus of all trampoline and drumhead devices on the chip containing device l will be the same, namely $\rho = 7.5 \times \rho_\text{g}$ and $Y = 110$ N m^{-1}.

Membrane Heat Capacity. The heat capacity C can be estimated from the mass density $\rho = 7.5 \times \rho_\text{g}$. The heat capacity for the GNB is

$$C = (c_\text{g} + 6.5 c_\text{p})\rho_\text{g} \times \text{a}^2$$

where a^2 is the device area (typically ~ 25 - 50 μm^2), c_g = 700 J kg^{-1} K^{-1} is the specific heat of graphene, and c_p ~ 1500 J kg^{-1} K^{-1} is the specific heat of PMMA. Together, the heat capacity for device l is $C \sim 4 \times 10^{-13}$ J K^{-1}.

Thermal Resistance. We can determine the thermal resistance R_T experimentally from the measured τ_T and C with the expression:

$$R_\text{T} = \frac{\tau_\text{T}}{C} \tag{6}$$

For the data shown in Figure 3c (device e), τ_T = 20 μs and $C \sim 2 \times 10^{-13}$ J K^{-1} gives R_T ~ 9.8×10^7 K W^{-1}. For the device l (Supplementary Figure 2), τ_T = 230 ns and $C \sim 4 \times 10^{-13}$ J K^{-1} gives R_T ~ 0.57×10^6 K W^{-1}.

Frequency-shift Responsivity. Experimental values for Y, σ_0, C obtained from electromechanical measurements together with τ_T obtained from off-resonant bandwidth measurements can be used to calculate the frequency-shift responsivity, which for a circular drumhead is given by

$$R_f = \frac{Y\,\alpha}{2\,\sigma_0(1-\nu)}\frac{\tau_\mathrm{T}}{C} \tag{7}$$

For the drumhead device l, τ_T = 230 ns and $C{\sim}4\times10^{-13}$ J K^{-1}. Using $\alpha = -7.4\times10^{-6}$ K^{-1} (ref. [18]) and the Poisson ratio for graphene $\nu = 0.16$, we calculate a frequency responsivity of $R_f{\sim}$ 2900 W^{-1}, which agrees within 10% of the independently measured value from frequency shifts measurements $R_f{\sim}2600$ W^{-1}, also for Device l.

For the trampoline device e, we use the heat capacity and mass density measured from the gate dependence of device l. However, we expect that the initial stress on the device was changed during the focused ion beam shaping. To estimate the initial stress more accurately, we use finite element analysis to determine the amount of initial stress required in a trampoline geometry to reproduce the measured $f_0 = 10.7$ MHz, which we find to be $\sigma_0 = 0.19$ N m^{-1}. Using these values along with measured τ_T = 20 μs and $C{\sim}2\times10^{-13}$ J K^{-1} (and assuming this model can be applied to trampolines), Supplementary Equation 7 gives $R_f{\sim}$ 250000 W^{-1}, which reasonably close to the directly measured value of $R_f{\sim}180000$ W^{-1}.

Optical Absorption Estimated from Mechanical Properties. Using the above frequency responsivity (Supplementary Equation 7) together with measured values of Δf_0, f_0, and the incident power (P_inc) allows for a calculation of the optical absorption coefficient

$$\beta \equiv \frac{\Delta f_0}{f_0\,R_f P_\mathrm{inc}}$$

For the drumhead device l and trampoline device e, we find $\beta = 2\%$ and $\beta = 1.6\%$, respectively. These values agree well with expected absorption for monolayer graphene[19,20] $\beta = 2.3\%$. The absorption obtained from cavity modeling was $\beta = 0.6\%$ but the mass density shows that the graphene is likely coated with contaminants, perhaps PMMA residue or amorphous carbon. This residue will absorb some light, which would raise β above 0.6%, closer to the measured 2.0%. To quote the most conservative value for the noise equivalent power, we use the highest estimate for the absorption at $\beta = 2.3\%$ for our calculation in the main text.

Supplementary Note 8.

Graphene Temperature. The temperature change of the GNB can be inferred from Fourier's Law

$$\Delta T = P_\mathrm{abs}R_\mathrm{T} = \beta P_\mathrm{inc}R_\mathrm{T} \tag{9}$$

where β is the optical absorption. Using the relation for absorbed power, $P_\mathrm{abs} = \Delta f_0/(f_0 R_f)$, the temperatures change can also be written

$$\Delta T = \frac{\Delta f_0}{f_0 R_f} R_\mathrm{T}$$

or

$$\Delta T = \frac{\Delta f_0}{f_0} \frac{2\sigma_0(1-\nu)}{\alpha Y} \tag{10}$$

When we illuminated trampoline device e (see Supplementary Figure 1) with high incident power, $P_\mathrm{inc} = 400$ μW, we observed the resonance frequency increase from 10.7 MHz to 35 MHz. According to Supplementary Equation 10 for this device and these experimental conditions, $\Delta T \sim 890$ K. Using Supplementary Equation 9 yields a similar, consistent value of $\Delta T \sim 920$ K. Assuming the silicon oxide boundary of the device is a thermal sink at fixed at room temperature (293 K), the temperature of the graphene membrane was ~ 1200 K. The high temperature of the GNB during this experiment did not damage the GNB device in any measurable way. We also note that the GNB continued to operate at this high temperature. However, this high temperature probably annealed some of the PMMA contaminating mass as the resonant frequency returned to a higher value after cooling back to room temperature.

Supplementary Equation 9 can be used to estimate the temperature change due to a line-width frequency shift:

$$\Delta T = \frac{1}{Q} \frac{2\sigma_0(1-\nu)}{\alpha Y}$$

We have substituted $\frac{\Delta f_0}{f_0} = Q^{-1}$, where $Q$is the quality factor. For devices in this work $Q \sim 1000$. With $\sigma_0 = 0.1$ N m^{-1}, $\nu = 0.16$, $\alpha = -7.4 \times 10^{-6}$ K^{-1} (ref. [18]), and $Y = 110$ N m^{-1}, we calculate $\Delta T \sim 200$ mK.

Minimum Detectable Temperature. The minimum detectable temperature of the resonator is defined as

$$\eta_\mathrm{T} = \frac{\sigma_f \sqrt{t}}{\left|\frac{df}{dT}\right|}$$

According the thermal Ohm's Law, $\Delta T = T - T_0 = P_\mathrm{abs} R_\mathrm{T}$, $df/dT \propto df/dP_\mathrm{abs}$. Therefore, df/dT is constant because df/dP_abs is constant (see Fig. 2b in main text). So,

$$\frac{df}{dT} = \frac{\Delta f_0}{\Delta T} = \frac{\Delta f_0}{P_\mathrm{abs} R_\mathrm{T}} = \frac{f_0 \Delta f_0}{f_0 P_\mathrm{abs} R_\mathrm{T}} = \frac{f_0 R_f}{R_\mathrm{T}}$$

and

$$\eta_\mathrm{T} = \frac{\sigma_f \sqrt{t}}{f_0 R_f} R_\mathrm{T}$$

or

$$\eta_{\mathrm{T}} = \eta R_{\mathrm{T}} \tag{8}$$

where $\eta = \frac{\sigma_f \sqrt{t}}{f_0 R_f}$ is the noise-equivalent power. Using $R_{\mathrm{T}} \sim 0.57 \times 10^6$ K W^{-1} calculated using Supplementary Equation 6 and the measured $\eta = 1500$ pW Hz$^{-1/2}$ for the drumhead device l (Supplementary Figure 2), Supplementary Equation 8 gives $\eta_T = 0.8$ mK Hz$^{-1/2}$. For the trampoline device e, with $R_T \sim 1 \times 10^8$ K W^{-1} and $\eta = 20$ pW Hz$^{-1/2}$, we calculate $\eta_{\mathrm{T}} = 1.1$ mK Hz$^{-1/2}$.

Using the modelling from Supplementary Note 2, we rewrite the minimum detectable temperature as

$$\eta_{\mathrm{T}} = \sigma_{\mathrm{A}} \sqrt{t} \frac{2\,\sigma_0 (1-\nu)}{\alpha Y}$$

we see that η_{T} is independent of device dimensions. In agreement with this prediction, the calculated η_{T} for all the devices using $\eta_{\mathrm{T}} = \eta R_{\mathrm{T}}$ has a narrow distribution $\eta_{\mathrm{T}} = 1.2 \pm 0.7$ mK Hz$^{-1/2}$, despite variations in geometry, noise, initial strain, etc.

Upper incident power limit of the GNB. The upper range of incident power of the GNB can be estimated from the temperature stability of graphene, $T_{\mathrm{S}} \sim 3000$ K (ref. [21]), and the expression $\Delta T = \beta P_{\mathrm{inc}} R_{\mathrm{T}}$. We approximate the upper power range

$$P_{\mathrm{inc}}^{\mathrm{u}} \sim \frac{T_{\mathrm{S}}}{\beta R_{\mathrm{T}}}$$

For the trampoline device e (see Supplementary Figure 2), we use the inferred $\beta = 1.6\%$ and $R_{\mathrm{T}} = 9.8 \times 10^7$ K W^{-1} and estimate $P_{\mathrm{inc}}^{\mathrm{u}} \sim 1.9$ mW. The maximum power input for drumhead will be higher. For the drumhead device l, we use the inferred $\beta = 2.0\%$ and $R_{\mathrm{T}} \sim 0.57 \times 10^6$ K W^{-1}, and estimate $P_{\mathrm{inc}}^{\mathrm{u}} \sim 263$ mW.

Temperature Fluctuations. Fundamentally, fluctuations in temperature[22] will limit the noise-equivalent power

$$\eta_{\mathrm{TF}} = \sqrt{\frac{4\, k_{\mathrm{B}}\, T^2}{R_{\mathrm{T}}}}$$

These temperature fluctuations arise from the quantized nature of energy exchange between the bolometer and the environment. For trampoline device e, $R_{\mathrm{T}} \sim 10^8$ K W^{-1} and $\eta_{\mathrm{TF}} \sim 223$ fW Hz$^{-1/2}$, The measured noise-equivalent power for device e is about $100 \times$ greater than η_{TF}. Currently our GNBs are still quite far from the fundamental fluctuation limit, and are instead limited by sources of frequency noise.

Supplementary References

1. Bunch, J. S. *et al.* Electromechanical Resonators from Graphene Sheets. *Science* **315**,

490–493 (2007).

2. Davidovikj, D. *et al.* Visualizing the Motion of Graphene Nanodrums. *Nano Lett.* **16**, 2768–2773 (2016).

3. Albrecht, T. R., Grütter, P., Horne, D. & Rugar, D. Frequency modulation detection using high-Q cantilevers for enhanced force microscope sensitivity. *J. Appl. Phys.* **69**, 668–673 (1991).

4. Allan, D. W. Statistics of Atomic Frequency Standards. *Proc. IEEE* **54**, 221–230 (1966).

5. Yoon, D., Son, Y.-W. & Cheong, H. Negative Thermal Expansion Coefficient of Graphene Measured by Raman Spectroscopy. *Nano Lett.* **11**, 3227–3231 (2011).

6. Zhang, X. C., Myers, E. B., Sader, J. E. & Roukes, M. L. Nanomechanical torsional resonators for frequency-shift infrared thermal sensing. *Nano Lett.* **13**, 1528–1534 (2013).

7. Taylor, J. R. *Classical Mechanics*. (2005).

8. Dolleman, R. J. *et al.* Optomechanics for thermal characterization of suspended graphene. *Phys. Rev. B* **96**, 165421 (2017).

9. Metzger, C., Favero, I., Ortlieb, A. & Karrai, K. Optical self cooling of a deformable Fabry-Perot cavity in the classical limit. *Phys. Rev. B* **78**, 035309 (2008).

10. Skidmore, G. D., Han, C. J. & Li, C. Uncooled microbolometers at DRS and elsewhere through 2013. *Proc. SPIE* **9100**, 910003 (2014).

11. Laurent, L., Yon, J.-J., Moulet, J.-S., Roukes, M. & Duraffourg, L. 12-μm-Pitch Electromechanical Resonator for Thermal Sensing. *Phys. Rev. Appl.* **9**, 024016 (2018).

12. Barton, R. A. *et al.* High, size-dependent quality factor in an array of graphene mechanical resonators. *Nano Lett.* **11**, 1232–1236 (2011).

13. Endoh, T. *et al.* Uncooled infrared detector with 12μm pixel pitch video graphics array. *Proc. SPIE* **8704**, 87041G (2013).

14. Mizrahi, U. *et al.* Large-format 17μm high-end VO x μ-bolometer infrared detector. *Proc. SPIE* **8704**, 87041H (2013).

15. Saleh, B. E. A. & Teich, M. C. *Fundamentals of Photonics*. (2012).

16. Barton, R. A. *et al.* Photothermal self-oscillation and laser cooling of graphene optomechanical systems. *Nano Lett.* **12**, 4681–4686 (2012).

17. De Alba, R. *et al.* Temperature-dependence of stress and elasticity in wet-transferred graphene membranes. *J. Appl. Phys.* **123**, 095109 (2018).

18. Chen, C. *et al.* Performance of monolayer graphene nanomechanical resonators with electrical readout. *Nat. Nanotechnol.* **4**, 861–867 (2009).

19. Dawlaty, J. M. *et al.* Measurement of the optical absorption spectra of epitaxial graphene from terahertz to visible. *Appl. Phys. Lett.* **93**, 131905 (2008).

20. Nair, R. R. *et al.* Fine Structure Constant Defines Visual Transparency of Graphene. *Science* **320**, 1308 (2008).

21. Kim, K. *et al.* High-temperature stability of suspended single-layer graphene. *Phys. Status Solidi - Rapid Res. Lett.* **4**, 302–304 (2010).

22. Richards, P. L. Bolometers for infrared and millimeter waves. *J. Appl. Phys.* **76**, 1 (1994).
